# Study-based evaluation of the Abbott RealTime High Risk HPV test in comparison to the HC2 HR HPV test in women aged ≥30 years using residual LBC ThinPrep specimens

**DOI:** 10.1186/s12879-016-1994-0

**Published:** 2016-11-11

**Authors:** Thomas Iftner, Lisa Wang, Angelika Iftner, Barbara Holz, Juliane Haedicke-Jarboui, Nathalie Iftner, Reinhard von Wasielewski, Peter Martus, Gerd Boehmer

**Affiliations:** 1 Division of Experimental Virology, Institute of Medical Virology, University Hospital Tübingen, Elfriede-Aulhorn-Str. 6, Tübingen, 72076 Germany; 2 Clinical Epidemiology and Applied Biometry, University Hospital Tübingen, Tübingen, Germany; 3 Amedes Laboratory Bad Münder, Bad Münder, Germany

**Keywords:** Abbott RealTime High Risk HPV, Hybrid Capture, HPV, Cervical cancer screening

## Abstract

**Background:**

High-risk human papillomavirus (HR HPV) testing is already part of cervical cancer screening programs in a number of countries. New tests need to be validated not only in clinical studies but also in routine screening settings with regard to their clinical performance.

**Methods:**

The Abbott RealTime High Risk HPV Test (RT hrHPV test) was evaluated in a random sample of 1,456 patients from a German routine screening population of 13,372 women ≥30 years of age screened primarily by liquid-based cytology (LBC) that was complemented by 48 CIN3+ cases. Clinical sensitivities, relative specificities and positive predictive values (PPV) for both HPV tests were determined based on histologically confirmed high-grade cervical disease (CIN3+) as clinical outcome.

**Results:**

HR HPV prevalence in residual LBC samples was found to be 5.4 % by the RT hrHPV test and 5.6 % by the HR HC2 test, respectively. The Kappa-value for overall agreement between the RT hrHPV test and the HC2 assay for detection of HR HPV was 0.87. Relative sensitivities for detection of CIN3+ in patients with abnormal cytology was 93.8 % for the RT hrHPV assay and 97.9 % for HC2 (*p*-value = 0.5). Relative specificities and PPVs were comparable for both tests. The highest PPV was calculated for the specific detection of HPV16 by the RT hrHPV test (84.2 %). The RT hrHPV test showed a reduced sensitivity for detection of HVP31-positive CIN3 + .

**Conclusion:**

The RT hrHPV assay is as sensitive and specific in detecting severe cervical lesions in women with abnormal cytology as the HC2 HR HPV test.

## Background

Since the introduction of opportunistic cytological screening in Germany in 1971 the cervical cancer mortality rate has notably decreased [1]. However, 4,600 new cases and approximately 1,500 deaths due to cervical cancer are diagnosed in Germany each year [2]. Moreover, 150,000 cases of cervical cancer precursors (CIN3) are detected annually [3] and cervical cancer is the cause for 1.5 % of all female cancer deaths in Germany [2]. As persistent infection with High-Risk Human Papillomaviruses (HR HPV) is a necessary risk factor for the development of (pre)-cancer, numerous HPV tests are nowadays commercially available [4] to be used in cervical cancer screening programs [5]. However, only a small number of these tests have been approved by the FDA [6] with the Digene Hybrid Capture 2 High-Risk HPV DNA test as the first one (HC2; QIAGEN Hilden, Germany). The HC2 has been developed for the collective detection of 13 carcinogenic HPV types (16, 18, 31, 33, 35, 39, 45, 51, 52, 56, 58, 59, 68) [7]. It is one of the best validated HPV tests whose methodology is based on nucleic acid hybridization with signal amplification for qualitative detection of HPV-DNA within cervical samples.

The Abbott Real*Time* High Risk HPV Test (RT hrHPV) is another fully automated HPV DNA test which is based on real-time PCR that targets the L1 region of the 13 carcinogenic HPV types 16, 18, 31, 33, 35, 39, 45, 51, 52, 56, 58, 59, 68 and for detection of HPV 66. The multiplex design of the assay allows HPV16 and 18 genotyping as well as the collective detection of the 12 remaining HPV types [8]. The RT hrHPV test has been fully validated by several cross-sectional studies that evaluated its clinical performance in referral populations [9–18]. However, to date only four screening population-based cross sectional studies [19–22] and one follow-up study are available [23]. The objective of this retrospective study was the cross-sectional evaluation of the RT hrHPV assay in a routine cervical cancer screening population comprising women aged ≥ 30 years in Germany.

## Methods

The study cohort and methodology has previously been published [24].

### Study design

**Fig. 1:**
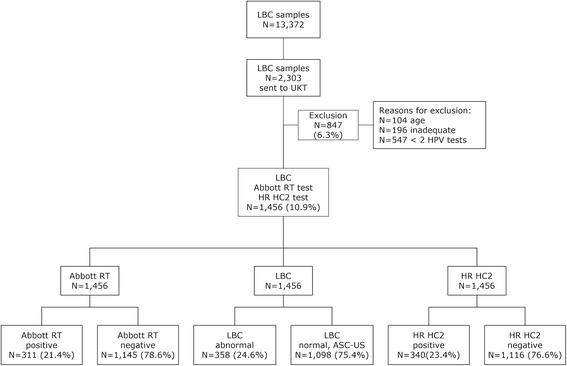
Study Flow Chart

Briefly the study was conducted on a sample of 2,303 women of a routine screening population of 13,372 women ≥ 30 years of age living in the Hannover area of Germany in 2011 (Fig. 1). Cervical samples were collected in PreservCyt® Pap Test specimen collection medium (Hologic) and cytology was tested within 1 week after collection in a central services laboratory (Amedes, Bad Münder, Germany). After exclusion of ineligible samples, a total of 1,456 residual liquid based cytology (LBC) smears from this cohort including all samples with abnormal and ASC-US cytology results as well as 10 % of randomly selected samples with normal cytology results were tested by both HR HC2 and Abbott RealTime High Risk HPV Test (RT hrHPV). This collection was complemented with samples from 48 patients with CIN3+ from a separate referral cohort in order to obtain a sufficient CIN3+ rate. HC2 tests were conducted in the laboratory of the Division of Experimental Virology at the University Hospital in Tübingen. RT hrHPV assays were performed in the Amedes laboratory within 2 weeks after collection. Samples with discordant HPV test results were genotyped by INNO-LiPA HPV Genotyping Extra® for discrepancy analysis.

Samples were anonymized thus, participants were unaware of their HPV test results and colposcopy as well as histopathology was only performed when indicated by German standard operating procedures. Samples with a primary histology of ≥ CIN2+ were independently reviewed by up to two pathologists.

### Sample collection

Sample Collection has previously been described [24]. Briefly cervical samples were collected in LBC PreservCyt® Collection medium (Hologic) using the Cervex broom according to routine guidelines. Samples were then centrally tested by cytology within one week of collection.

### Liquid based cytology

LBC was performed as described before [24] by the ThinPrep® 2000 Processor (Hologic) following the manufacturer’s instructions. Cytology results were reported using the Munich Nomenclature II and were translated into The Bethesda System (TBS) ([25]; Table 1). LBC results were considered negative when the result was Pap I/II (Normal) or Pap IIw (ASC-US); all other results were considered positive [24].

**Table 1:** HPV prevalence detected by the RT hrHPV test and the HC2 test in comparison to the liquid based cytology (LBC) results

LBC	RT + ve % (95 % CI)	HR HC2 + ve % (95 % CI)	κ (95 % CI)
Normal (Pap I/II)	5.4 % (4.2-6.9)	5.6 % (4.3-7.1)	0.78 (0.69-0.87)
ASC-US (Pap IIw)	21.4 % (12.7-33.8)	28.6 % (18.4-41.5)	0.62 (0.38-0.86)
ASC-H, AGC (Pap III)	50.0 % (28.0–72.0)	50.0 % (28.0–72.0)	1 (1.0–1.0)
LSIL, HSIL (Pap IIID)	65.1 % (59.6–70.3)	72.1 % (66.8–76.9)	0.76 (0.68–0.84)
HSIL, CIS (Pap IVa)	94.7 % (82.7–98.6)	100 % (90.8–100)	1
HSIL, CIS, Micro (IVb)	100 % (34.2–100)	100 % (34.2–100)	1
Micro, Invasive (PapV)	100 % (20.6–100)	100 % (20.6–100)	1
HSIL+ (≥Pap IVa)	95.1 % (83.9–98.7)	100 % (91.4–100)	1
AGC+ (≥Pap III)	68.0 % (62.9–72.5)	74.3 % (69.5–78.6)	0.78 (0.70–0.84)
Overall	21.4 % (19.3–23.5)	23.4 % (21.3–25.6)	0.87 (0.84–0.90)

+ve: positive; −ve: negative; +: and worse

95 % CI for HPV prevalence were calculated using the Wilson Score method

### HPV testing and genotyping

LBC samples were tested by Abbott RealTime High Risk HPV Test (RT hrHPV) in compliance with the manufacturer’s instructions. All specimens included in this study were initially tested by RT hrHPV test and subsequently analyzed by Digene Hybrid Capture 2 High-Risk HPV DNA (HC2) test. HC2 testing has previously been described [24].

HPV genotyping was carried out using the INNO-LiPA HPV Genotyping Extra® test (Fujirebio LiPA Extra) which identifies 20 HPV genotypes classified as Group 1, 2A and 2B carcinogens (16, 18, 26, 31, 33, 35, 39, 45, 51, 52, 53, 56, 58, 59, 66, 68, 69, 70, 73, 82) and 8 low-risk HPV or intermediate risk genotypes (6, 11, 40, 43, 44, 54, 71, 74). LiPA Extra is a line blot assay based on SPF-10-PCR as described previously [26]. Strips were scanned and analyzed automatically with a flatbed scanner and the LiRAS software (Fujirebio).

### Histology reviews

As detailed before all samples with an initial histology result of ≥ CIN2+ were reviewed by an independent external expert. In the case of a discrepant review reading, a second histology review was performed. If two out of three diagnoses were identical, the result was considered final [24].

### Statistical analysis

As described [24] statistical analysis was performed on all samples with valid LBC, RT hrHPV and HC2 (N = 1,456) test results. To calculate the agreement between RT hrHPV and HC2, the Cohen’s kappa value (κ) was used. The Wilson score method was used to calculate 95 % confidence intervals (CI) for HPV prevalence. Moreover, clinical sensitivity and relative specificity as well as positive predictive values (PPV) and negative predictive values (NPV) were calculated according to Cuzick et al. [20] based CIN3+ histology results.

Relative performance of the two tests was measured by calculating the ratio of the sensitivity to the specificity. This ratio of the sensitivity of the two tests was defined as the True Positive rate of the first test divided by the True Positive rate of the second test. The relative specificity on the other hand, is dependent of prevalence, and was expressed as Spec(RT hrHPV)/Spec(HC2) = (0.818-Prevalence)/(0.800 - Prevalence). The delta method was used to determine confidence intervals. A full description of this method has previously been published [24]. Statistical analysis of the relative sensitivity and specificity was calculated using the statistics software package R version 3.0.2.

## Results

Of 2,303 overall specimens a total of 847 samples had to be excluded from the analysis either because they returned unsatisfactory cytology results (*n* = 196), because they could not be analyzed by all three tests due to insufficient material (*n* = 547) or because study participants were younger than 30 years of age (*n* = 104). The 1,456 remaining specimens were tested by both RT hrHPV and HC2 (Fig. 1). 358 of these samples had an abnormal cytology result (Pap ≥ III; ≥ ASC-H) 56 were classified as ASC-US (Pap IIw) and 1,042 women had normal cytology results (Pap I/II) (Table 1 and 2). Colposcopy and histopathology on biopsies was only performed on 33 patients with abnormal cytology results independent of the HPV test results. A total of 9 CIN2 cases were identified by review histopathology. The remaining 24 biopsies were < CIN2. In order to obtain a higher CIN3+ rate, a total of 48 CIN3+ cases were added to the cohort.

**Table 2:** Overall RT hrHPV and HR HC2 test results

		HR HC2		
		Positive (N/%)	Negative (N/%)	Total (N/%)
RT	Positive	293/20.1 %	18/1.2 %	311/21.4 %
	Negative	47/3.3 %	1,098/75.4 %	1,145/78.6 %
	Total	340/23.4 %	1,116/76.6 %	1,456/100 %

*HR HC2* Digene Hybrid Capture 2 High-Risk HPV DNA test, *RT* Abbott RealTime High Risk HPV Test

### HPV prevalence and type distribution

**Fig. 2:**
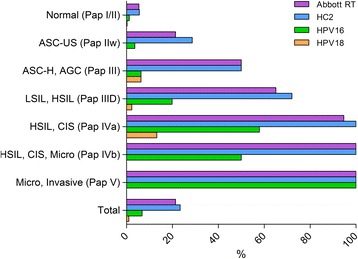
Prevalence of HR HPV detected by HC2 and RT; and HPV 16 and HPV 18 genotyping by RT

Concordant HPV DNA test results were obtained from 1,391 of 1,456 LBC samples (Table 2). Thus the overall percentage of agreement was 95.5 % and the Kappa coefficient was κ = 0.87 (95%CI: 0.84-0.90). The overall HR HPV positivity rate determined by HC2 was high with 23.4 % and 21.4 % by RT hrHPV. In the cytology normal group (Pap I/II) HR HPV was detected in 5.4 % of the specimens by RT hrHPV and in 5.6 % using the HC2 assay, respectively (Table 1 and Fig. 2). In the ASC-US category, HPV prevalence was 21.4 % for RT hrHPV and 28.6 % for HC2. In samples with abnormal cytology HR HPV was detected in 50 % of the LBC specimens classified as ASC-H, AGC (Pap III) by RT hrHPV as well as HC2, compared to 65.1 % versus 72.1 %, respectively, in the LSIL, HSIL (Pap IIID) category and 94.7 % (RT hrHPV) versus 100 % (HC2) in category HSIL, CIS (Pap IVa). HPV prevalence in women with glandular abnormal cytology (Pap III+; AGC+) was 68 % detected with RT hrHPV and 71.3 % with HC2 test.

Cohen’s Kappa Coefficients (κ) were calculated to measure the agreement of RT hrHPV and HC2 within the different LBC categories (Table 1). Kappa values (κ) were excellent for normal (Pap I/II) samples (κ = 0.78) and all abnormal LBC categories ≥ LSIL (≥Pap III κ = 0.78). For ASC-US (Pap IIw) specimens the agreement was fair (κ = 0.62).

A total of 65 discordant samples were detected between the RT hrHPV and HC2 (Table 3). 47 discrepant samples (72.3 %) were RT hrHPV-negative and HC2-positive compared to only 18 samples (27.7 %) with HC2-negative and RT hrHPV-positive test results. The majority of discordant results was detected in specimens with ASC-H (PapIII 14.3 %) cytology results followed by the LSIL, HSIL (Pap IIID, 10.3 %) and the normal (Pap I/II, 2.3 %) cytology categories. All deviant samples (*n* = 65) were genotyped using the INNO-LiPA HPV Genotyping Extra test in order to identify false positive or false negative samples (Table 3). As a result we resolved 5 RT hrHPV-negative and 7 HC2-negative samples as true negatives. While four samples were inadequate for LiPA genotyping, all remaining samples (*n* = 49) were HPV DNA positive.

**Table 3:** HPV genotyping of discordant samples with the LiPA Extra test

HPV Genotype	HPV classification	HC2-ve RT + ve (N)	RT-ve HC2 + ve (N)	Histology CIN3+ (HC2-ve/RT-ve)
HPV 16^a^	HR	2	1	0
HPV 18^a^	HR	0	3	0
HPV 31^a^	HR	0	6	0/2
HPV 33^a^	HR	0	0	0
HPV 39^a^	HR	0	1	0
HPV 45^a^	HR	2	0	0
HPV 51^a^	HR	4	3	0
HPV 52^a^	HR	0	4	0
HPV 56^a^	HR	0	1	0
HPV 58^a^	HR	0	1	0
HPV 59^a^	HR	1	0	0
HPV 68^a^	HR	0	0	0
HPV 53	Intermediate	0	11	0
HPV 66^b^	Intermediate	0	1	0
HPV 70	Intermediate	0	2	0
HPV 6	LR	0	1	0
HPV 54	LR	0	1	0
HPV 74		1	1	0
HPVX		0	2	0
HPV DNA -ve		7	5	0
no result (sample failed)		1	3	0
Total		18	47	0/2

^a^ HC2 and RT target types; ^b^ RT target type; *HC2*, Digene Hybrid Capture 2 High-Risk HPV DNA test, *RT*, Abbott RealTime High Risk HPV Test, *HPVX* HPV DNA was detected by LiPA, but could not be correlated to a specific type; *HR* High-risk, *LR* Low-risk; +ve: positive; −ve: negative; +: and worse

In detail one sample missed by HC2 contained a non-target type of the HC2 assay and a total of 9 specimens (50 %) were false-negative by HC2. However, none of the samples missed by HC2 had a histology result of CIN3+. Interestingly, the HC2 assay detected HPV DNA in 18 RT hrHPV-negative samples, which contained non-target types of either HPV DNA test including 11 cases of HPV 53.

In contrast 18 discordant samples (38.3 %) with negative RT hrHPV and positive HC2 test results were non-target types of the RT hrHPV test (including 2 HPVX types), while another 21 samples (44.7 %) were false-negative by RT hrHPV revealing an unusually high false-negative rate for the RT hrHPV test. These 21 samples also included two specimens with CIN3+ histology. Both samples were found to be positive for high risk HPV type 31 by LiPA Extra genotyping with a total of six CIN3+ samples positive for this HPV type.

**Fig. 3:**
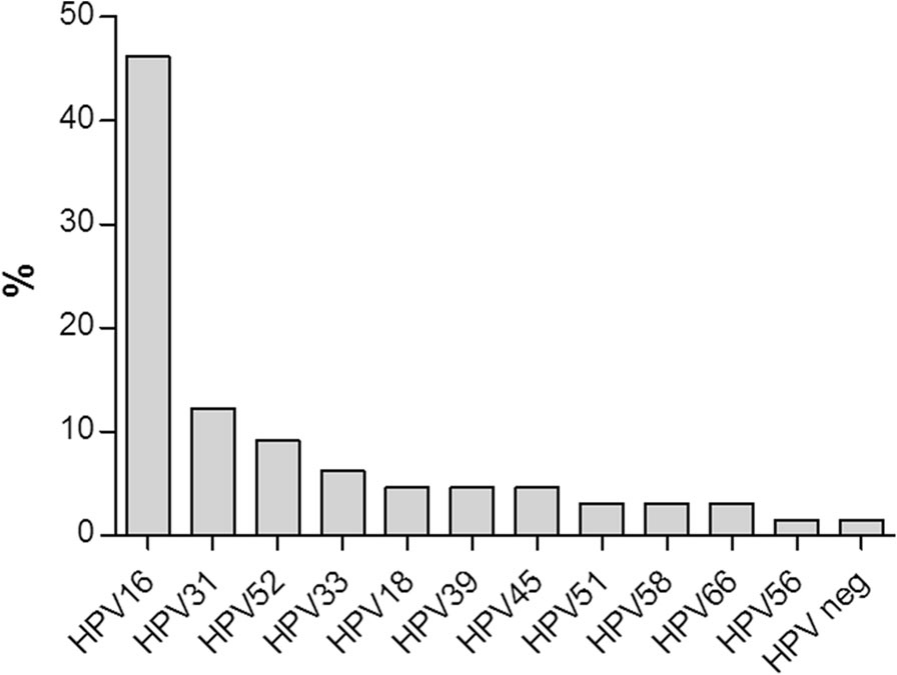
HPV genotype distribution in specimens with high grade cervical disease (CIN3+)

The RT hrHPV test concurrently detects 14 different HPV types and generates genotyping information for specific identification of the highly carcinogenic types HPV 16 and 18. The association of HPV 16 and HPV 18 with different cytology outcome as detected by RT hrHPV is shown in Fig. 2. The overall detection rate for HPV 16 (6.8 %) was higher than that of HPV 18 (1.0 %). Prevalence of HPV 16 and 18 increases proportionally with higher grade of cytology abnormalities and was highest in HSIL and CIS (≥Pap IVa). 58.5 % of these samples were positive for HPV 16 and 12.2 % for HPV 18. In contrast HPV 16 prevalence was 2.2 % compared to 0.2 % for HPV18 in the normal (Pap I/II) and 3.6 % versus 0 %, respectively, in the ASC-US (Pap IIw) category. HPV 16 and HPV 18 were both detected in 6.3 % of the specimens with ASC-H, AGC (Pap III) cytology results in contrast to the LSIL, HSIL (Pap IIID) category, which included 19.9 % HPV 16 positive and 2.3 % HPV 18 positive samples. All samples with a histology result of CIN3+ were genotyped by using the INNO-LiPA HPV Genotyping Extra test. Overall HR HPV genotype distribution in specimens with CIN3+ lesions is shown in Fig. 3. All HPV types detected in both single and multiple infections were included in the calculations. Not surprisingly, HPV 16 (46.2 %) was the most prevalent genotype in specimens with CIN3+ lesions followed by HPV 31 (12.3 %), HPV 52 (9.2 %), HPV 33 (6.2 %) and HPV 18 (4.6 %). All specimens with a LiPA Extra genotyping result of either HPV 16 or HPV18 were correctly identified by the concurrent Abbott RT HPV 16/18 genotyping.

### Sensitivity and specificity

Relative sensitivity and specificity were calculated to define the diagnostic accuracy of the HR HC2 and the RT hrHPV test for detection of CIN3+. Within this cohort 81 biopsies were available from patients with abnormal cytology results. Overall relative sensitivity for detection of CIN3+ in patients with abnormal cytology was 93.8 % for the Abbott RealTime hrHPV assay and 97.9 % for HC2, a difference which is insignificant (*p*-value = 0.5; Table 4). Similar results were obtained for the endpoint CIN2+ (94.7 % vs 98.2 % respectively). Relative specificities and positive predictive values (PPV) were comparable 81.1 % and 16.9 % for RT hrHPV and 79.8 % and 16.5 % for HC2, respectively. The PPV for the detection of CIN2+ was highest at 84.2 % for the separate detection of HPV 16 by RT hrHPV. Overall 50 histology reviews were performed. Sensitivity and specificity results for the performances of both HPV tests with endpoint CIN3+ did not change when the review results were accounted for in the respective calculations (data not shown).

**Table 4:** Test characteristics of the RT hrHPV and HC2 tests for detection of high grade cervical disease (CIN3+)

	Relative sensitivity CIN2+		Relative sensitivity CIN3+		Relative specificity <CIN2		PPV CIN2+		NPV < CIN2	
	%	95 % CI	%	95 % CI	%	95 % CI	%	95 % CI	%	95 % CI
HR HC2	98.2	89.4–99.9	97.9	97.5–99.9	79.8	77.5–81.8	16.5	12.8–21.0	99.9	99.4–100
Abbott RT	94.7	84.5–98.6	93.8	81.8–98.4	81.1	78.9–83.1	16.9	13.1–21.6	99.7	99.2–99.9
Abbott RT HPV16	56.1	42.4–66.9	60.4	45.3–73.9	75.0	52.9–89.4	84.2	59.4–68.1	41.9	27.4–57.8
Abbott RT HPV18	7.0	2.3–17.8	6.3	1.6–18.2	77.5	75.2–79.6	50	17.4–82.6	27.4	17.9–39.3
Abbott RT non 16/18	33.3	21.7–47.2	29.2	17.4–44.3	54.2	33.2–73.8	63.3	43.9–79.5	25.5	14.8–39.9

+: and worse; *CI* Confidence interval

**Fig. 4:**
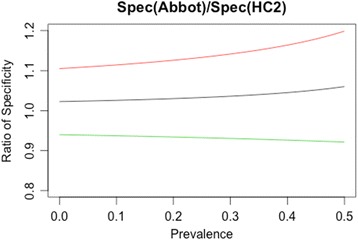
Specificity ratio of RT hrHPV and HC2 as a function of HR HPV prevalence (green line: lower confidence interval boundary; red line: upper boundary)

Colposcopy was given only to women with abnormal cytology findings; therefore the true number of high grade CIN in the normal (Pap I/II) and ASC-US (Pap IIw) categories is not known. Due to this lack of definitive data one could not calculate the clinical sensitivity and specificity of the two tests. However, we calculated the ratio of the sensitivity and specificity of the two tests to each other, thus we are able to determine relative, if not absolute, performance. The ratio of sensitivities between RT hrHPV test and HC2 is 0.959 (95%CI: 0.885–1.033). Fig. 4 shows the ratio of specificity as a function of prevalence. We found no statistical difference between clinical sensitivity and specificity of the RT hrHPV test and HC2 for the detection of high grade disease at the 95 % confidence level.

## Discussion

This study was conducted to evaluate the analytical and relative performance of the RT hrHPV test using residual LBC specimens selected from a German routine cervical cancer screening population. HR HPV positivity rates overall (23.4 % for HC2 and 21.4 % for RT hrHPV) and in LBC normal samples (5.4 % for RT hrHPV and 5.6 % for HC2) were in line with previously published data [21]. HPV prevalence in women with abnormal cytology (AGC+ ≥ PapIII) was 68 % detected by RT and 71.3 % for the HC2 test. The generally slightly higher detection rates of the HC2 test may be attributed to cross-reactivity with non-target types [27]. The most prevalent HPV type in women with high grade cervical disease in this study was HPV 16, which is in accordance with all recent studies and meta-analyses (summarized by [28]).

Comparing the performances of both tests we showed that the RT hrHPV and HC2 test performed similarly and the agreement of both assays was excellent (κ = 0.87). These results reflect previously reported data [13, 19, 21] and indicate that both HPV DNA tests performed equivalently. Discordant samples were analyzed by the LiPA HPV genotyping Extra test, which has been used as an adjudicating assay in test comparison studies before [24, 29, 30], due to its high analytical sensitivity [31]. We found that 50 % of HC2-negative, but RT hrHPV-positive samples were true HPV negative. The possibility of false-negative HC2 results has previously been reported to be attributed to a low viral copy number leading to false-negative HC2 results [32] or to the lack of an internal control for cellularity, which is however ruled out here by the split sample protocol. Furthermore, we found that the HC2 test was able to detect infections with the non-target type HPV 53. Cross-reactivity of the HC2 test with HPV 53 has been demonstrated by multiple previous reports. In fact, HPV 53 has been shown to be one of the most frequent non-target types detected by the HC2 test through cross-hybridization of its HR probe [21, 33–35]. While the HC2 test detected all cases of CIN3+, the RT hrHPV test missed two CIN3+ cases positive for HPV 31. Similar results have previously been published by Poljak et al., who reported that the RT hrHPV test missed two CIN3 + −cases with HPV 31 and 58 co-infections [36]. Two other reports also demonstrated a diminished sensitivity of the RT hrHPV test for HPV 31-positive CIN3 cases [21, 30]. Indeed in the present study we found that one of the two CIN3+ cases missed by RT hrHPV test represented a co-infection with HPV-type 33, which might indicate competitive primer binding in the PCR leading to an unfavorable kinetic of amplification for HPV 31 or other types from the alpha-9 subgenus in mixed infections.

Comparing concurrent genotyping results of the RT hrHPV test we were able to show that RT HPV 16/18 genotyping correctly identified all specimens with a LiPA Extra genotyping result of either HPV 16 or HPV 18. These results are in contrast with earlier reports showing concordance between LiPA and RT genotyping in only 90 % of the tested specimens [10].

Relative sensitivities for detection of CIN3+ were high and comparable for both tests (*p*-value = 0.5) whereas relative specificities for RT was slightly higher than for the HR HC2 test. By calculating the ratios of sensitivities and specificities, respectively, we were able to confirm that no statistical difference exists between the two tests’ performances. Our observations are in line with cross-sectional studies reported previously in routine screening populations [19–21] and suggest that RT hrHPV test is well suited to be used in routine primary cervical cancer screening or adjunctive to cytology. Further evidence for the applicability of the RT hrHPV test in primary screening has recently been published by Poljak et al., reporting the first longitudinal data for the Abbott RT HPV test [23]. The authors demonstrated the non-inferior clinical performance of the RT HPV test in a routine screening population of 3,920 women with a 3-year follow-up time in comparison to the performance of the HC2 test.

In summary we found that the sensitivities and relative specificities of the RT hrHPV and the HR HC2 test are comparable. However, it appears that the RT hrHPV test has a reduced sensitivity for the detection of HPV type 31, which in this study has led to two CIN3+ cases missed by the RT hrHPV test. Because HPV 31 is one of the most prevalent high-risk HPV-types worldwide [37] and is found in 3,5 % of global cervical cancer cases [38], it is important to carefully assess risks and benefits of applying the RT hrHPV test. A considerable benefit of the RT hrHPV HPV test is its separate type specific detection of the most prevalent HPV genotypes 16 and 18, which together account for a total of an estimated 70.4 % of cervical cancer cases worldwide [38]; and we demonstrated that the PPV for detecting high grade disease is by far highest for Abbott RT hrHPV HPV 16.

## Conclusions

We provided evidence that the Abbott HR HPV test is suitable for primary routine screening in Germany and other countries with a similar infrastructure regarding the secondary prevention of cervical cancer.

## Abbreviations

AGC: Atypical glandular cells
ASC-H: Atypical squamous cells – cannot exclude HSIL
ASC-US: Atypical squamous cells of undetermined significance
CI: Confidence interval
CIN: Cervical intraepithelial neoplasia
CIS: Carcinoma in situ
HC2: Digene Hybrid Capture 2 High-Risk HPV DNA Test
HPV: Human Papillomavirus
HR: High-risk
HSIL: High grade squamous intraepithelial lesion
LBC: Liquid-based cytology
LSIL: Low grade squamous intraepithelial lesion
NPV: Negative predictive value
PPV: positive predictive value
RLU/CO: Relative light units/cutoff
RT hrHPV test: Abbott RealTime High Risk HPV Test
